# Exploring the perceptions and experiences of community health workers using role identity theory

**DOI:** 10.3402/gha.v8.28045

**Published:** 2015-09-18

**Authors:** Langelihle Mlotshwa, Bronwyn Harris, Helen Schneider, Mosa Moshabela

**Affiliations:** 1 Rural and AIDS Development Action Research Programme (RADAR), School of Public Health, Faculty of Health Sciences, University of the Witwatersrand, Johannesburg, South Africa; 2 Centre for Health Policy/MRC Health Policy Research Group, School of Public Health, Faculty of Health Sciences, University of the Witwatersrand, Johannesburg, South Africa; 3 School of Public Health, University of the Western Cape, Cape Town, South Africa; 4 MRC/UWC Health Services to Systems Research Unit, Bellville, South Africa; 5 Discipline of Rural Health, School of Nursing and Public Health, University of KwaZulu-Natal, Durban, South Africa

**Keywords:** community health worker, role identity theory, home-based care

## Abstract

**Background:**

Community health workers (CHWs) are an integral resource in many health systems, particularly in resource-poor settings. Their identities – ‘who’ they are – play an important role in their hiring, training, and retention. We explore the perceptions, experiences, and identities of CHWs as they adopt a CHW role in rural South Africa, using ‘role identity theory’.

**Design:**

From April to December 2010, we conducted 18 semi-structured interviews with CHWs volunteering in non-governmental home-based care (HBC) organisations in one rural sub-district in South Africa. The role identity theory framework was used to understand the work of CHWs within their communities, addressing themes, such as entry into, and nature of, caring roles, organisational support, state resourcing, and community acceptability. A thematic content analysis was used to analyse the collected data.

**Results:**

The study found that CHWs usually begin their ‘caring work’ before they formally join HBC organisations, by caring for children, neighbours, mothers, fathers, friends, and the community in some way. CHWs felt that becoming a health worker provided an elevated status within the community, but that it often led community members to believe they were able to control resources. The key role identities assumed by CHWs, as they sought to meet patients’ and their own needs, were a complex mix of community ‘insider’, ‘outsider’, and ‘broker’. Each of these role identities served as a unique way to position, from the CHW's perspective, themselves and the community, given the diversity of needs and expectations.

**Conclusions:**

These role identities reveal the tensions CHWs face as ‘insider’ members of the community and yet at times being treated as ‘outsiders’, who might be regarded with suspicion, and at the same time, appreciated for the resources that they might possess. Understanding role identities, and how best to support them, may contribute to strategies of retention and sustainability of CHW programmes, as their formalisation in different contexts continues to grow.

Globally, community health workers (CHWs) are recognised as an important resource in addressing the critical shortage of human resources for health (1). CHWs are volunteers who manage patients at the community level, reside within the community, and may receive a stipend for the work they do (2). They have limited training, mainly in service, often provided by the Department of Health as well as other organisations (3). Their services continue to receive international attention as part of the response to HIV/AIDS and weakened health systems in resource-poor settings (4–6). The World Health Organization has suggested that CHWs, through the provision of diverse services, including offering communities a link to the broader healthcare system, preventative care, and keeping track of disease outbreaks, can improve health outcomes in communities (7).

South Africa has recently re-prioritised the importance of primary health care (PHC) and addressing the social determinants of health (8). CHWs have been accorded a central role within the district health system, as members of teams promoting PHC and community-based health services (9–11). Yet despite many studies on CHWs and the work they do (12), few have examined ‘who’ CHWs are and how their identity in this light might influence why and how they adopt and perform their work (1, 13).

Several studies have noted that CHWs take on a number of ‘formal’ roles as part of ‘extending a helping hand’ to the health system and communities themselves: education (health information); community outreach; supporting ‘high-risk’ individuals, including orphans and vulnerable children; administering treatment for different illnesses, for example, tuberculosis (TB) and HIV/AIDS; and identifying and referring maternal cases for ante- and post-natal care (13, 14). Additionally, they are commonly involved in informal, yet important activities such as visiting neighbours to talk to them about problems they could be facing, cooking, and fetching water for community members who cannot do it for themselves, and dealing with family feuds (3). However, these activities are rarely recognised as community health work by the health system (15).

Many of these ‘less visible’ roles of CHWs result from the multifaceted nature of their work, which necessitates some degree of flexibility and adaptability to different situations and for different patients (16). Additionally, CHWs often face challenges such as managing stigma, caring for patients who cannot afford to buy food, lack of resources such as diapers for bedridden patients, and inadequate training to care for patients with complex needs (17–19). Such complex demands challenge who they are and their work, and may lead to feelings of role inadequacy (13, 17, 20). Conversely, as suggested by studies elsewhere, including in South Africa, a sensitive induction process, regular in-service training, and a supportive organisational context may generate a positive CHW role identity, supporting and teaching CHWs as they learn how to care for themselves and others with different challenges (21, 22).

There is, however, a risk that, if not carefully managed, the adoption of a CHW role may generate an identity of superiority, associated with professions (here, a ‘medical role’), which distance them from their communities (14), and may lead to unintended negative consequences for the implementation of CHW programmes.

This paper seeks to gain insight into perceptions and experiences of CHWs adopting and enacting CHW roles in rural South Africa, using the role identity theory (23). Recognising how and why these roles are adopted and practiced is critical in promoting CHW retention, intervention efficacy, recruitment, and programme success (14).

## Conceptual framework

Farmer et al. (24) define role identity as the process through which individuals give meaning to themselves in relation to a specific role. Role identity theory seeks to explain how roles develop in societies and how people represent ‘who’ they are in different contexts, including their working environment (25). Roles can be seen as a set of relationships; the feedback CHWs receive in any particular role relationship shapes the meaning they make and the identities they construct around that role (in turn re-shaping those roles).

Roles and identities are fluid and flexible, and through enactment, are constantly renegotiated based on the nature of feedback received. This is especially so for CHWs who occupy an ambiguous position as simultaneous representative of community and of the health system. Moreover, these roles are far less defined and codified than those of formalised professionals, and becoming a CHW thus involves an active process of relational, role, and identity ‘work’ (26). How others respond to an individual occupying a particular role, as well as their own expectations and motivations, will influence their perceptions about the role and its relationship to identity; ‘who’ they perceive themselves to be (14).

Role identity theory also suggests that the process of becoming a CHW is socially moulded by the belief system in which CHWs are located, as well as their previous experiences, individual beliefs, and social surroundings (26, 27). Thus, adopting the role of a CHW means culture, behaviour, and cognitive processes influence the way they organise and shape their work, and how they respond within their working environment.

## Methods

### Study design

We conducted in-depth, semi-structured interviews with 18 CHWs as part of a 3-year mixed method study exploring quality of care and support of home-based care (HBC) in rural Bushbuckridge, South Africa (28, 29).

### Study setting

Bushbuckridge is a rural area located in Mpumalanga province in the north-east of South Africa. The area is underdeveloped and characterised by high unemployment, poverty, disease, and poor service delivery (30, 31). Because of poverty in the area, many people depend on social grants received from the government, most particularly child support, old age, and disability grants (32). The area is served by three hospitals, two community health centres, 37 clinics, and five mobile clinics, which provide services to a population of more than 500,000. Although located near the Kruger National Park, a popular tourist destination, the local community has limited involvement and economic benefits (31). A census conducted at the time of data collection (2010) counted a total of 943 CHWs volunteering in 37 HBC organisations in the area (28).

### Study population and sampling

From the 37 organisations in the area, 9 were purposively selected to ensure a diverse representation of the HBC organisations (29). From each of the nine organisations, two CHW were selected, also using a purposive criterion-based sampling method to ensure a wide range of representation: sex, age, education level, village they work in, length of time working as a CHW, length of time working for the organisation, whether the CHW received a stipend or was a volunteer, type of care provided, and number and type of patients served by each CHW.

### Data collection

Semi-structured interviews were carried out between April and December 2010 by three teams, each comprised a researcher and trained field worker. The interview guides were in English and translated to the local languages in the area: Northern Sotho, XiTsonga, and SiSwati. These were piloted and adapted prior to adoption in the study. Interviews took place in a language preferred by the participant and at a location where they were comfortable, for example in a private area at the clinic. For purposes of clarity and to explore additional themes, eight follow-up interviews – guided by vignettes – were conducted. The vignette for each participant was drawn from their primary interview and told as a hypothetical story, about an individual caring for people within a community, which served as a prompt and encouragement for the participant to begin to speak about their experiences. Interviews took between 20 and 60 min. Interviews were translated and transcribed verbatim, and validated by a different researcher.

### Data analysis

A thematic content analysis was used to analyse the data (33, 34). Interviews were analysed manually, drawing (deductively) on role identity theory, and (inductively) from participants’ stories. Three researchers systematically generated codes, which were developed into sub-themes and themes. Another researcher validated the thematic codes against the original data and between the different coders. If any codes were inconsistent or lacking in agreement, consensus was reached by the researchers after reanalysing the data. Table 1 below shows examples of how themes and sub-themes were developed.

**Table 1:** Examples of moving from text to themes

Phrases		Codes	Subthemes	Themes
1.	I was born here in 1949. I have grown up here and started to help people …	Being born and grown up in the community, starting to help people	Roots embedded in the community	A sense of belonging: the insider role identity
2.	I live here in the area, I am Doreen. I am married and God has blessed me with a boy and a boy …	Living and being married in the area, being blessed with two boys		
1.	We trained for two months and two weeks then we came back for practical's then we went back to school to write our final exam …	Getting formal training, doing practical, going back to school, having formal exams	Assuming a professional status	Looking in: an outsider role identity
2.	They trained us about home-based care and they also run workshops here …	Being trained in home- based care, participating in workshops		
1.	We also call an ambulance to the hospital or clinic to take the patient	Referring patients, calling ambulances	Mediating access to health care and social services	Standing in-between: a broker role identity
2.	And I also helped the orphans to get food parcels	Participating in social development		

### Ethics

All participants gave their written informed consent before the beginning of the interview. The University of the Witwatersrand and Mpumalanga Provincial Department of Health granted ethical clearance for the study to be conducted. Participants are referred to in the findings by pseudonyms.

## Results

### Profile of participants

All but two of the participants (16 of 18) were women and the majority (12 of 18) were aged between 25 and 44 years, with four participants older than 60 years. Nearly, half (8 of 18) had been working in their organisations for 5–7 years. All of the participants had received some formal education, with one-third completing secondary school (5 of 12) or beyond (1 of 18), and the older participants reporting some Adult Basic Education and Training (ABET) (Table 2).

Fifteen participants described receiving some form of CHW-related training at the start of, and/or during, their employment as CHWs, with most exposed to training in more than one area of care (Table 2). Just over half (10 of 18) of the participants received stipends of ZAR500 or ZAR1000 (US$ 50 or US$ 100) per month, with the rest considered unpaid volunteers.

**Table 2:** CHW education levels and on the job training

CHW education, *N=*18 (%)		CHW training, *N=*18 (%)	
≥Primary school	3 (17)	DOT, TB	9 (50)
ABET	3 (17)	HIV/AIDS	8 (44)
Some secondary school	6 (33)	Voluntary Counselling and Testing (VCT)	6 (33)
Grade 12	5 (28)	Home-based care	5 (28)
Beyond Grade 12	1 (6)	Ancillary health care	2 (11)
		Old age	1 (6)
		Peer education	2 (11)
		First aid	3 (17)
		None	2 (11)

DOT, TB: directly observed treatment for tuberculosis.

The interpretation of the results is presented under the headings of the themes and sub-themes with quotations to show how the analysis is rooted in the participants’ perceptions and experiences.

During the interviews, we explored why participants became CHWs – that is, adopted a CHW ‘role’ – and what being in this role, namely being a CHW, meant to them.

### A caring role: becoming a CHW

Caring for, and educating, community members about their health was presented as key to both becoming and being a CHW. Participants were largely motivated by altruism and commitment to serving their communities. Most portrayed their decision to take on this role as a proactive and positive choice.

This is my destiny to help my community. Since 2005 I have been volunteering until now in 2010. This shows how determined I am, even if I do not get paid I want to help the community which I love …. [Doreen, Female, 28]

The motivation to become a CHW was often explained through previous personal experiences as patients, and themselves being cared for by a CHW while sick. In a way they saw becoming a CHW as an opportunity for ‘giving back’.

I was a patient also, when I got really sick, these grannies [CHWs] told me about the support group at the HBC organization and urged me to come, and I went to it. I noticed that people are surviving …. When I got better, I asked if I could join, and she agreed that I could be a CHW. This is how I started to work and counsel others who were like me …. [Busi, Female, 31]

Many mentioned practicing a caring role, long before becoming a CHW, for example, by caring for relatives or friends, and transitioning into a CHW role seemed natural. Formal participation in the HBC system was simply an extension of this wider ‘carer’ role, or an additional way to demonstrate care for others and the community. For them, there was continuity between a non-formal caring experience and a formal caring experience as a CHW.

Before this I was taking care of my mother. There was a child who was suffering from Asthma and I was also taking care of that child because they were staying together with my mother. During that time, the experience inspired me to help others who are suffering from different illnesses …. [Dethapelo, Female, 37]

However, some participants reported that lack of education or employment, rather than altruism or a caring identity *per se*, had forced them to embrace this caring role. In the Bushbuckridge area, it is generally difficult to get any form of employment, and some thus felt pushed into volunteering in the hope that this would lead to a job elsewhere. These participants displayed some desperation but felt this caring ‘job’ was better than doing nothing:

I want to be a social worker and that means caring for the needy …. So I thought maybe I should try HBC as I am sitting at home doing nothing …. [Phumzile, Female, 22]

[The reason] is that I didn't have another work to do, since I am not educated and I have realized that I can do the work that I am doing today, even if I am not educated and it is possible for me to do it …. [Noria, Female, 35]

The caring role identity was however, sometimes challenged by misconceptions of the CHW motivations, and they believed that this led to community members not accepting them and their work.

#### Fear of breaching confidentiality (mistrust)

Despite CHWs coming from within the community, and believing themselves to be caring, some felt they were not always entirely trusted or welcome in homes, which they attributed to fears they would violate patient confidentiality. The tension that exists with the job of being a CHW – being both a caregiver and ordinary neighbour – was highlighted in some accounts.

Some would refuse to be our patients …. [Thembi, Female, 40]

You find out that when you are neighbours you do not trust each other and you find that it is difficult for neighbours to share their problems …. [Dethapelo, Female, 37]

In particular, they felt high levels of stigmatisation of HIV/AIDS was a hindrance to their work, and contributed to the community lack of trust, as their work was often (at times exclusively) associated with nursing those living with HIV. Many reported that the biggest fear for clients was that of being gossiped about within the community, that people would point fingers at them, saying that they could have the ‘disease’ (HIV) if they were seen to be visited by a CHW.

Esther disclosed her [HIV] status to me, and she told me that she would not like me to come to her place […]. So we agreed on how we would visit each other [more privately] …. [Olga, Female, 60]

### A sense of belonging: the insider role identity

All of the CHWs interviewed were born or had married into the communities in which they were working and living. Constructing their working identities as insiders seemed to reinforce their sense of belonging and lent purpose to their daily tasks, which were linked to the close relationships they had within the community and a sense that assisting one another was ‘the right thing to do’. The notion of starting from within, of belonging and working as an insider, was further exemplified by some participants who had not only adopted a CHW role as individuals but were the founders of HBC organisations – mostly women who saw a need within the community.

#### Roots embedded in the community

Central to the narratives of most participants was the notion that being part of the community was more than being a member of a geographic space, but rather, understanding people's needs and helping where required. This, they suggested, had prepared them for the work that they did. As they were insiders of the community, they learnt to take care of each other before adopting a formal CHW role.

Before all that I used to work for my brother. I baby sat his child and did other chores at his home. After that I came back here and became a caregiver at a home-based care in [name of organization] …. [Faith, Female, 29]

#### Active in community structures

Some participants spoke about actively supporting and participating in community structures, forums, and programmes that serve the purpose of community development. Church was a place of interaction, contributing to a sense of belonging in their particular community. Additionally, religion played an important role in their narratives through its emphasis on the need for caring for others ‘as God would expect of them’, an expression of a religious calling to service. This often translated into their daily practices as CHWs and ways of interacting with patients:

I come from a family of prayer. I'm a Christian which is why I understand going around caring for people who are sick. To every household I go to, I offer them prayer …. [Nicoline, Female, 60]

#### Ability to identify as ordinary community members

Many also commented that their daily challenges as community members, as well as CHWs, were similar to those faced by their patients, for example, lack of money for food and other basic commodities. Minimal stipends were received by just more than half the participants, with the rest working as volunteers. Doreen, one of the participants, explained how difficult community health work could be as she had no money to assist her patients who also had no money. Similarly, Dorcas reiterated this by explaining how CHWs do not have food to give their patients, yet wished they had.

Sometimes if one of our patients is sick I was suggesting that maybe if we can give them food because some of them are sick and don't have anything to eat, so if we were providing them with food I think it would be much better. [Right now] We can only go and greet the patients then do health talks, that is it …. [Dorcas, Female, 27]

However, she also, like Olga, felt that such shared hardship allowed a common understanding and co-creation of experience with her patients and, for both women this generated a sense of spatial and social connection within the community.

You have to share that little amount you are earning, to give a client to buy some bread and at some point, we used to give some soups to the patients from our own families to help them, just because we have a conscience, they do not have food and we cannot report it to our seniors as they will tell you that there is no food to give to the clients …. Sometimes we do not have …. [Olga, Female, 30]

### Looking in: an outsider role identity

#### Assuming a professional status

CHWs are, by definition, recognised within the health system and by community members, for their health knowledge and training. This identity comes with certain expectations and, although participants identified closely as community ‘insiders’, many felt that simply ‘being’ a CHW served to elevate their status within the community to teachers, social workers, and doctors:

They see me as though I am a doctor and they are satisfied with the care I give the patient …. [Mahlatsi, Female, 72]

This was because many CHWs felt that they could teach about different health issues (particularly TB and HIV/AIDS which seemed to be the most talked about, and stigmatised, health conditions), as well as sometimes connect their patients to social services. Although this transformation from ‘ordinary community member’ to ‘professional’ was often a source of pride, CHWs also acknowledged that they felt it created a distance between them and the community members. Being a CHW meant they did not always belong; they became ‘outsiders’, although this was beyond their control or intention.

#### Spying for the government

Participants described difficulties trying to balance their duties with both the community and the professional health sector, and do so without compromising their credibility with both clients and health workers. They sometimes felt like they were seen as government spies who reported on community members, leading to refusal of CHW assistance.

The problem is they (the patients) start hating me when they don't receive the grant anymore. They think that when I go to them to get a report on how they are feeling and I take this information to [name of institution or facility] so that they can cancel the grant because they are feeling well. They hate me when their grant gets cut off …. [Jennifer, Female, 43]

However, many of the participants explained how they did all in their ability to advocate for social welfare, which they felt was expected from them in their role as CHWs in the community.

### Standing in-between: a broker role identity

The efforts CHWs made in trying to balance both insider and outsider roles proved challenging for many but sometimes allowed the adoption of an alternative identity: that of the broker.

#### Mediating access to health care and social services

The ‘middle man’ or brokering role was often most visible when CHWs were able to connect patients with needed resources and services – whether within the health system (such as referrals to clinics and hospitals) or beyond (including schools, social and development services, and appropriate non-governmental organisations).

I also helped a child who passed his matric [grade 12 or final year of secondary school] without his ID document. What I did, I told him to go to the primary school and to the high school to collect letters that he was attending there and he did as I told him to do. Then we went to the police station and the Home Affairs offices and I explained that he does not have an ID document and the birth certificate …. [Gloria, Female, 60]

#### Brokering limited by lack of resources

For many CHWs, the role of brokering between communities and the health system was also limited by a lack of resources. Weak organisational contexts and/or unsupportive health services made it difficult for some of the CHWs to be as effective as they felt they could be. Although some participants used their own resources to assist their patients, others made do with ‘alternative’ improvised materials to assist in cleaning or bathing their bedridden patients. Some CHWs explained they did not have anything besides the psychosocial support which they offered the patients, in an attempt to emphasise the only resource available to them.

Instead of gloves we just use plastics for bread [bread packets] while waiting for them to give us the kit. You find that they say there is no money to buy the kit [the HBC kit contains the gloves] …. [Jennifer, Female, 43]

I can't lie, there is nothing they do [referring to the organization] …. [Dorcas, Female, 27]

This brokering role entailed CHWs having to use their own (limited) personal resources to assist patients despite themselves living with the challenges of poverty. Some of the CHWs explained that they did not have anything besides the psychosocial support which they offered their patients – a brokering of solidarity.

## Discussion

This exploratory study provides insights into the importance of identity amongst CHWs, how they see themselves and perceive the health system and society to see them, as well as how this influences their provision of services for patients within their communities. Role identity theory teaches that in a role – here, as a CHW – a person responds to their environment and makes choices that seem appropriate to the particular situations, sometimes shifting from one role identity to another, in an attempt to make sense of the contradictions and challenges faced (35). In first becoming and then maintaining their roles, the CHWs in the study had to constantly negotiate and re-negotiate their identity. These role identities were seemingly conflicted, but also potentially complementary, and included community insiders working from within, outsiders working from outside but looking in, and brokers standing in-between the community and the health system to facilitate health care and social services.

The insider identity prevented the CHW status from being labelled as something different, special, or ‘more’. The CHW role was framed around that of ‘mere’ volunteer work carried out in service of community. Preparation and ‘training’ of CHWs came largely from their personal experiences as patients and of caring for others, and not necessarily a professional background. Yet, because of this proximity, they were able to identify with other patients, from both sociocultural and socioeconomic standpoints. This also suggests that some of their motivation came from altruism (21, 36, 37) as they felt they understood how people within the community needed this assistance. In this regard, it could be seen as embodying the spirit of ‘ubuntu’, or humanness and kindness, as a prevalent social norm in rural African communities (38). The work of CHWs was also motivated by the fact that they came from impoverished communities with little access to employment and other work opportunities. The adoption of a CHW role enabled some degree of repositioning within their own communities (39), while was for some the taking of a developmental role, a potential path out of the conditions that they and their patients had to endure.

As participants described their interactions with different community members, it was clear that identifying as an ordinary community member was not always possible. CHWs thus also identified as community outsiders, as being in some way different from their patients. Their role as health workers, with specialised technical knowledge and in certain instances privileged access to sensitive information about individual community members, gave them an elevated status and a measure of acceptance in communities (39, 40). This, however, also created a professional distance between them and their patients, and their association with HIV/AIDS care, and a perceived ‘surveillance’ function, at times led to their rejection, a pattern found elsewhere (40). The gendered nature of CHW work provided a special opportunity for women to accumulate respect, recognition, and a good reputation (39), but at the same time reinforced gender norms that place the burden of low-paid and often volunteer caring work on the shoulders of women (18, 41, 42).

Despite struggling to care for patients, many CHWs were prepared to go to great lengths to fulfil their roles, and as described elsewhere, often at some personal cost (18, 29, 43). In many ways, maintaining positive role identities was closely tied to the third, brokering, role, which involved being able to confront an interrelated set of material and other challenges in getting the job done, including contributing their own resources (material and psychosocial), while being able to tap into institutional and system support. Where the brokering role is adequately encouraged and supported, CHWs can become a significant voice for communities (22, 44, 45), and conversely, act as health system representatives within communities (46).

### Limitations

This study was qualitative in nature, localised to a rural South African setting. Participants may have exaggerated their responses to present themselves in the best possible light, potentially resulting in social desirability bias. The choice of sampling (purposive) sought a range of CHWs, from different backgrounds, experience, and organisations, although potentially involved researcher subjectivity and bias in the selection of participants. In addition, the sample is not necessarily a representation of the population of CHWs in the area. Longitudinal designs in future research can be used to better understand the unfolding patterns of identity formation over time, and as the policy and health system context evolves.

## Conclusion

In this study, we noted the importance of the process of becoming a CHW, how this work does not begin when recruited by the health system but rather emerges from pre-existing roles as members of communities, how it fits with social norms of caring, but also with acceptable spaces of individual advancement. These supportive environments can be used as tools and entry points to better provide resources for their work and enable the brokering role.

As the health system continues to integrate CHWs formally in South Africa, it is central that they acknowledge the perspective of care given by CHWs according to their personal experiences. The importance of placing their work between the formal health system and civic society will allow policy reforms to develop broader systems that acknowledge this care economy.

CHWs are important for strengthening the health system as well as improving health service delivery at the grassroots level (47). Given the heavy reliance on CHWs especially in rural and remote areas in caring for people in the home environment, the state, non-governmental organisations, and communities will need to develop innovative ideas in retaining this cadre for a long-term period. The state's attitude towards CHWs in policy reforms of PHC is central. Locating them firmly as part of community members who are neighbours, friends, or spouses allows them to gel and be who they are – ‘ordinary community members’. Although still in this position the health system awards them a status of being more than community members, allowing them to teach, promote health, and become health system representatives supported with resources. However, their community roles can be undermined when they are perceived to be playing policing roles for the formal health system and determining resource allocations or conducting health surveillance functions. Efforts in this regard must be made to allay community fears and explain the value of household-level screening and data gathering by CHWs that form part of their formalised roles. The inherent tensions in their work associated with caring for stigmatised conditions such as HIV/AIDS need to be addressed as does issues of confidentiality. More generally, there is a need to further explore what it means to be a CHW and to nurture a sense of belonging for this cadre, given that CHWs face unique, potentially isolating challenges by being both inside and outside of the communities to which they are so closely tied, personally and professionally. Finally, there is a need for instrumental/material support for community acceptance and legitimacy of CHW work. This will thus impact positively in CHW programmes ultimately improving PHC.
